# Socioemotional Competencies of Primary Education Teachers and Their Relationship with Attitudes Toward Inclusive Education

**DOI:** 10.3390/bs16040539

**Published:** 2026-04-03

**Authors:** Miguel Ángel Albalá Genol, Claudia Messina Albarenque, Talía Gómez Yepes, Edgardo Etchezahar

**Affiliations:** Departamento Interfacultativo de Psicología Evolutiva y de la Educación, Facultad de Formación del Profesorado y Educación, Universidad Autónoma de Madrid, Campus Cantoblanco, C/Francisco Tomás y Valiente, 3, 28049 Madrid, Spain; miguel.albala@uam.es (M.Á.A.G.); claudia.messina@uam.es (C.M.A.); talia.gomezy@uam.es (T.G.Y.)

**Keywords:** inclusive education, social competence, primary education, teacher attitude, emotional development

## Abstract

In recent years, inclusive education in Spain has undergone significant legislative and pedagogical progress. However, its implementation still faces challenges, particularly regarding teachers’ attitudes and practices. The main objective of this study was to analyze how socioemotional competencies (self-esteem, prosociality, and emotional autonomy) are related to primary school teachers’ attitudes toward inclusive education. A total of 590 teachers from the Valencian Community, aged between 25 and 60 years (Mage = 33.33 years; 88.7% women), participated in the study by completing a battery of validated quantitative scales. The results indicated that most teachers exhibited medium levels across all three competencies, with significant differences by gender (higher prosociality in women and higher self-esteem in men) and teaching experience (prosociality decreased over time, while self-esteem increased). Furthermore, a positive and significant relationship was found between the three socioemotional competencies and inclusive attitudes, with emotional autonomy emerging as the strongest predictor, followed by self-esteem and, to a lesser extent, prosociality. These findings underscore the need to incorporate socioemotional competence development into teacher training programs to foster more inclusive educational environments. The implications and limitations of the relationships between the examined socioemotional competencies, inclusive attitudes, and sociodemographic and professional experience variables are discussed.

## 1. Introduction

At present, inclusive education continues to pose a major challenge for educational systems, as its effective implementation is essential to address and eradicate issues such as exclusion, segregation, and the inequality experienced by students. Since inclusive education began to be promoted in Spain, numerous changes have been introduced with the aim of improving the inclusion of all students. In Spain, the rigorous implementation of what is currently understood as inclusive education began in the 1990s with the enactment of Organic Law 3/1990 of 3 October on the General Organization of the Education System (LOGSE). This legislation was the first in Spain to propose the integration of special education into the mainstream educational system as a means of reducing segregation, as well as to introduce the term “students with special educational needs,” which has been maintained in all subsequent educational laws (García-Rubio, 2017; Gómez Yepes et al., 2023, 2024). Since then, various institutional actions have been developed to promote inclusive education, including specific initiatives aimed at helping students overcome the barriers that limit their presence, participation, and achievement.

According to the most recent report issued by the General Council of Psychology in Spain, in recent academic years the proportion of students assessed as having specific educational needs has increased by up to 17% (Ministry of Education, Vocational Training and Sports, 2025). This increase indicates that approximately 12% of the total student population currently presents specific educational support needs (ACNEAE). Of this group, 25.9% (292,897 students) have special educational needs associated with some type of disability or severe disorder, while the remaining 72.8% (704,192 students) present other needs derived from situations of socio-educational vulnerability. According to the Ministry of Education, Vocational Training and Sports (2025), this group of students—representing 3.6% of all students enrolled in non-university education—is predominantly educated in mainstream schools (85.2%), while the remaining 14.8% attend special education schools.

### 1.1. Teachers’ Attitudes Toward Inclusive Education

In Spain, studies show that teachers’ attitudes toward inclusive education are not homogeneous, with positions that are sometimes poorly defined or even neutral regarding students with special educational needs and learning difficulties (Padilla, 2021; Tárraga et al., 2025). In this context, teachers play a crucial role in the development of inclusive education, making it highly important to examine their attitudes toward its implementation as well as other related competencies (Sevilla et al., 2018). Attitudes toward inclusive education are defined as the cognitive, affective, and behavioral disposition that teachers display toward student diversity, directly influencing the implementation of inclusive practices in the classroom (Albalá Genol et al., 2023; Charitaki et al., 2024; Gómez Yepes et al., 2025). These attitudes may be positive or negative and are shaped by personal and contextual factors, such as the training received, previous experience with students with special educational needs, and institutional support. According to García Martínez and Checa Domene (2024), teachers’ attitudes toward diversity and inclusive education constitute a key factor in promoting an inclusive school that ensures learning opportunities for all students.

Thus, according to Tárraga et al. (2025), primary school teachers’ attitudes toward inclusive education are generally moderately positive, although they vary depending on personal, training-related, and contextual factors. This systematic review shows that while most studies report a favorable predisposition toward inclusion, ambiguous or neutral positions are also identified, especially when teachers perceive limitations in resources, institutional support, or specific training. More recent research conducted in the Spanish context (García Martínez & Checa Domene, 2024) confirms this trend and highlights that teachers’ attitudes toward inclusion in primary education are influenced by previous experience with students with special educational needs and by perceptions of professional self-efficacy.

In comparable international contexts, the literature agrees that primary teachers’ attitudes toward inclusive education constitute a multidimensional construct that is also sensitive to personal and professional variables. Accordingly, although primary teachers tend to display more inclusive attitudes than secondary teachers, their level of acceptance and commitment to inclusion varies depending on the type of student diversity and the emotional and organizational demands perceived in the classroom. Likewise, empirical research in the European context shows that favorable attitudes toward inclusion are associated with greater implementation of inclusive practices, whereas more ambivalent attitudes are linked to less adaptive pedagogical approaches (Charitaki et al., 2024).

Beyond the Spanish context, international research consistently highlights that the successful implementation of inclusive education depends not only on legislative frameworks but also on teachers’ beliefs, emotional preparedness, and perceived professional competence. Comparative studies conducted across European and non-European contexts indicate that, despite policy advances, teachers frequently report concerns related to insufficient training, lack of institutional support, and the emotional demands associated with addressing diverse learning needs (McGrath & Van Bergen, 2019).

In this regard, previous research has documented persistent structural and professional barriers to inclusion, including resource constraints, increased workload, emotional exhaustion, and uncertainty regarding effective pedagogical strategies (Candeias et al., 2021; Hettiarachchi & Das, 2014). Such challenges may contribute to ambivalent or less favorable attitudes toward inclusion over time, particularly when teachers perceive a mismatch between inclusive policy expectations and available support systems.

Therefore, it becomes evident that attitudes toward inclusive education are closely related to teachers’ socio-emotional competencies, also leading—within current educational contexts—to a greater willingness to respond to student diversity. These socio-emotional competencies act as a mediating factor between inclusive beliefs and teaching practice, fostering more equitable classroom climates and a more consistent implementation of inclusive education. In this regard, teachers’ socio-emotional development emerges as a key element in consolidating sustainable inclusive attitudes in primary education.

### 1.2. Teachers’ Socio-Emotional Competencies and the Relationship with Inclusive Education

Primary school teachers have always required a wide range of psycho-pedagogical skills, as they must be prepared to promote the development of a broad set of knowledge and basic competencies within educational communities. In this regard, various studies have established the defining principles of teachers committed to inclusive education (Echeita Sarrionandia et al., 2017; Hernández-Izquierdo & Marchesi, 2021): demonstrating a reflective disposition that continuously connects what they do with how they do it, as well as with the core values of inclusive education (equality, equity, respect for diversity, social justice, among others). In this sense, previous studies have shown a strong association between teachers’ personal and social competencies and favorable attitudes toward inclusive education (Rangel-Pacheco & Witte, 2023).

The structure of these competencies is rapidly changing alongside current technological development and societal transformation (Atabeki, 2020; Zhi et al., 2024). Educational theories have placed particular emphasis on the notion of key competencies, understood as sets of skills, knowledge, attitudes, and values that are essential for individuals’ personal development (Tahirsylaj & Sundberg, 2025). The selection and content of key competencies are based on socially accepted and shared values, which frame them as necessary for personal life and active participation in society (Ferreiro & Zabalza, 2021). Specifically, in the case of teachers, competencies for successful professional performance can be classified as follows (Lozano-Peña et al., 2021): professional competencies, necessary for pedagogical, psychological, and didactic preparation for teaching; performance competencies, referring to teachers’ physical and neuropsychological skills; personal competencies, encompassing appropriate social and personal characteristics of teachers; social competencies, related to teachers’ moral and ethical values as role models for students; and motivational competencies, linked to teachers’ identification with their professional role. Consequently, teachers’ personal and socio-emotional competencies are highly relevant in the current context for achieving inclusive professional development (Ungaretti et al., 2023).

The importance of teachers’ competencies for inclusive practice becomes evident in their impact on student learning (Albalá Genol et al., 2023; Gómez Yepes et al., 2025). Evidence shows that in increasingly heterogeneous classrooms requiring more inclusive practices, teachers often feel unprepared and concerned about their ability to cope with such demands (Molina & Ríos, 2010). Thus, a lack of specific knowledge and training in inclusive methodologies discourages early childhood and primary education teachers from supporting children with special educational needs and learning difficulties in their classrooms (Hettiarachchi & Das, 2014).

On this basis, a key element for ensuring inclusive education is the development of socio-emotional competencies (Padilla, 2021). Broadly defined, these competencies refer to individuals’ abilities to identify and regulate their emotions, as well as their social behavior (Pérez-González et al., 2020). According to Eisenberg et al. (2010), the socio-emotional competencies most strongly associated with teachers’ inclusive attitudes are prosociality, emotional autonomy, and self-esteem. First, emotional autonomy—understood as the capacity to feel, think, and make decisions independently—enables teachers to become their own “reference authority” (Mikulic et al., 2015). Emotional autonomy involves not only emotional awareness but also the capacity for independent regulation, self-determined decision-making, and resilience when facing uncertainty or external pressures (Mikulic et al., 2015). Previous research has highlighted that teachers’ emotional self-regulation and autonomy are closely associated with adaptive coping, reduced emotional exhaustion, and more effective inclusive practices (Lozano-Peña et al., 2021; Calandri et al., 2025). Moreover, recent reviews on teacher emotional competence in inclusive education contexts emphasize regulatory and resilience-related components as particularly critical for sustaining inclusive attitudes over time (Calandri et al., 2025). In this sense, it represents a balanced position between emotional dependence and affective detachment. Second, prosociality refers to voluntary actions carried out for the benefit of others, such as sharing, donating, caring, comforting, and helping (Colomeischi, 2015; Gómez Yepes et al., 2024; Sánchez-Pujalte et al., 2021). Finally, self-esteem is defined as self-evaluation that is congruent with one’s emotional state and with the emotional perceptions of significant others (Collie et al., 2015).

However, socio-emotional competencies do not appear to receive sufficient emphasis in teacher education programs (Benito Ambrona et al., 2022). In Spain, emotional education programs for teachers have been proposed (Pérez Escoda et al., 2013), but they have shown varying degrees of acceptance and implementation within in-service teacher training programs (Benito Ambrona et al., 2022). Some studies have examined the extent to which teacher education programs address topics relevant to socio-emotional development and its practical application. Nevertheless, these studies have consistently found that training programs devote little attention to the practical development of socio-emotional competencies among professionals and, consequently, among their students (Esteve, 2006). This situation may be related to the assumption that teachers, having already been deemed competent by default, do not require specific training in these skills. Another reason lies in the traditional prioritization of conventional aspects of teacher preparation (didactics, subject-matter knowledge, organization, and school curriculum, among others) at the expense of socio-emotional competencies. The traditional conception of teacher education—largely focused on teaching future professionals subject didactics and pedagogical and curricular aspects—should be revised and adapted to current educational contexts characterized by socio-educational diversity, where socio-emotional development competencies can have a significant impact on academic achievement. In this regard, recent studies highlight the importance of strengthening teachers’ socio-emotional competencies, as this enables them to better manage diverse classrooms and ensure inclusive education (Calandri et al., 2025).

**Figure 1:**
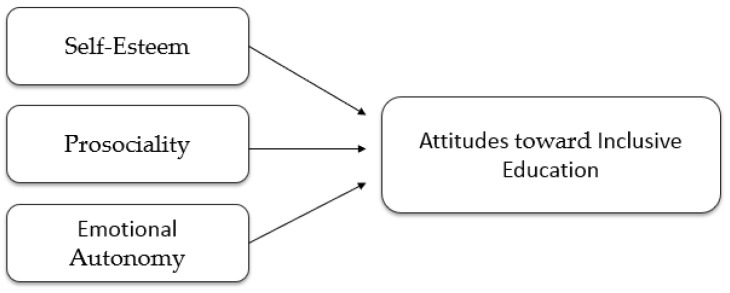
Conceptual model of the associations between socio-emotional competencies and attitudes toward inclusive education.

In conceptual terms, the three socio-emotional competencies examined in this study represent distinct yet complementary psychological resources that may differentially shape teachers’ attitudes toward inclusive education. Firstly, Self-esteem refers primarily to teachers’ global self-evaluation and perceived professional worth, which may influence their confidence in addressing diverse classroom needs and adopting inclusive practices. Secondly, Prosociality reflects an interpersonal orientation toward helping, empathy, and concern for others, potentially fostering greater openness to student diversity and supportive classroom interactions. Thirdly, Emotional Autonomy, in contrast, involves the capacity for independent emotional regulation, decision-making, and resilience under pressure, which may enable teachers to cope with the emotional demands and uncertainties associated with inclusive settings (Figure 1). From this perspective, while Prosociality may promote motivational willingness to support inclusion and self-esteem may strengthen perceived competence to do so, Emotional Autonomy may function as a regulatory mechanism that sustains positive inclusive attitudes when challenges arise. Accordingly, the present study examines the differential contribution of each of these competencies to teachers’ attitudes toward inclusive education.

Based on the above, the present study pursues two objectives: first, to analyze the levels of socio-emotional competencies (self-esteem, emotional autonomy, and prosociality) in a sample of primary school teachers; and second, to examine how each of these competencies is related to attitudes toward inclusive education.

## 2. Materials and Methods

### 2.1. Sample

The sample was non-probabilistic and purposive in nature and consisted of 590 primary school teachers residing in the Valencian Community (Spain), aged between 25 and 60 years (M = 33.33; SD = 9.22). Of the participants, 88.7% identified as female and 11.3% as male. Regarding professional experience, participants were distributed across different career stages: early-career teachers (0–3 years), mid-career teachers (4–19 years), and late-career teachers (20–36 years), allowing for comparative analyses based on years of service. All participants were actively teaching at the primary education level at the time of data collection.

### 2.2. Measures

An ad hoc questionnaire was developed, which included the following scales.

***Self-esteem:*** the Rosenberg (1965) Self-Esteem Scale was used, translated into Spanish by Echeburúa (1995) and previously applied to samples of teachers in the Spanish context (e.g., Brígido Mero & Borrachero Cortés, 2011). The scale consists of ten items assessing feelings of self-respect and self-acceptance. Half of the items are positively worded and the other half negatively worded. Responses are provided on a four-point Likert-type scale (1 = Strongly agree, 2 = Agree, 3 = Disagree, 4 = Strongly disagree). For scoring purposes, negatively worded items (3, 5, 8, 9, and 10) are reverse-coded, and all items are then summed. Total scores range from 10 to 40. The scale has shown high reliability indices, with test–retest correlations ranging from 0.82 to 0.88, and adequate levels of internal consistency assessed using Cronbach’s alpha (ranging from 0.77 to 0.88) (e.g., Tomaka et al., 1993). In a Spanish adult and adolescent clinical sample, Vázquez Morejón et al. (2004) found a strong correlation between self-esteem scores and total scores on different types of socio-emotional competencies (0.31 < r < 0.61). The Cronbach’s alpha in that study was 0.87, consistent with previous findings, and temporal reliability was acceptable both at two months (r = 0.72) and at one year (r = 0.74).

***Prosociality:*** the Prosocial Behavior Scale (PBS; Auné et al., 2016), adapted for teachers, was used. The PBS is a self-report measure designed to assess levels of prosociality as a stable trait in the adult population across four dimensions: Empathic Behaviors (EB), Altruism (AL), Helping (HE), and Sharing and Donating (SD). Previous studies have reported adequate psychometric properties for the scale (0.70 < α < 0.79). The instrument consists of 32 items with a six-point Likert-type response format reflecting the frequency of the behavior (1 = Never, 2 = Almost never, 3 = Sometimes, 4 = Often, 5 = Almost always, 6 = Always). The instructions present a generic and atemporal context for prosocial behaviors.

***Emotional autonomy:*** the Adult Emotional Development Questionnaire (CDE-A), developed by Pérez Escoda et al. (2013), was used to assess different dimensions of emotional competence. The instrument comprises 48 items rated on an 11-point Likert-type scale and allows for the identification of needs across five dimensions of emotional competence (Bisquerra Alzina & Pérez Escoda, 2007). For the purposes of this study, the Emotional Autonomy dimension was used. This subscale consists of eight items and has demonstrated adequate reliability and validity in previous studies with Spanish teachers.

***Attitudes toward inclusive education:*** attitudes toward inclusive education were assessed using the inventory developed by Unianu (2012) and de Boer et al. (2012), which empirically examines different dimensions of inclusive education, with particular emphasis on special educational needs. The assessment includes statements addressing the cognitive component of attitudes, comprising 12 items, as well as a “central perspectives” scale based on the “My Thinking About Inclusion” evaluation (Stoiber et al., 1998). To assess the affective component of attitudes, the Multidimensional Attitudes Toward Inclusive Education Scale (MATIES; Mahat, 2008) was used, focusing on teachers’ feelings of frustration and irritation. As the affective items primarily reflect negative emotions, additional items reflecting positive emotions such as confidence were incorporated from the “Competencies” questionnaire developed by Avramidis et al. (2000). Finally, the behavioral component of attitudes was assessed using the MATIES scale (Mahat, 2008), which examines teachers’ willingness to interact with and support students. Responses were recorded using a five-point Likert scale ranging from 1 (Strongly disagree) to 5 (Strongly agree). Higher scores on each dimension indicate more favorable attitudes toward inclusive education.

***Sociodemographic variables:*** questions were included to collect information on participants’ gender and age.

The selection of instruments was guided by three criteria: (a) prior validation in Spanish-speaking populations, particularly in educational or adult samples; (b) strong psychometric evidence of reliability and construct validity in previous research; and (c) conceptual alignment with the socio-emotional dimensions examined in this study and their documented relevance to inclusive education. Accordingly, widely used and empirically supported scales were chosen in order to ensure measurement comparability with the previous literature and methodological robustness.

### 2.3. Procedure and Data Analysis

Participants were invited to take part in the study on a voluntary basis, and informed consent was obtained. Participants were also informed that the data derived from this research would be used exclusively for scientific purposes, in accordance with the National Law on the Protection of Personal Data (Organic Law 3/2018), ensuring the anonymity of participants and guaranteeing compliance with the ethical guidelines proposed for the conduct of research in the Social Sciences and Humanities. Data analysis was performed using the statistical software SPSS version 22. In all cases, the assumptions of normality and homoscedasticity of the variables under study were tested. All procedures carried out in this study were in accordance with the ethical standards of the institutional and research committee and with the 1964 Declaration of Helsinki and its subsequent amendments or comparable ethical standards.

## 3. Results

### 3.1. Preliminary Analyses

Prior to conducting the main analyses, preliminary tests were performed to evaluate data quality and verify the assumptions required for parametric statistical procedures. Examination of skewness and kurtosis values indicated acceptable distributional properties for all variables (|skewness| < 1.20; |kurtosis| < 1.50). In addition, multicollinearity diagnostics showed tolerance values ranging from 0.61 to 0.79 and variance inflation factors (VIF) below 2.00, indicating the absence of problematic collinearity among predictors.

Table 1 presents descriptive statistics and Pearson correlation coefficients for all study variables. As shown, teachers reported moderate mean levels of self-esteem, prosociality, and emotional autonomy, as well as moderately favorable attitudes toward inclusive education.

Significant positive correlations were observed between all socioemotional competencies and attitudes toward inclusive education. Emotional autonomy showed the strongest association (r = 0.52, *p* < 0.001), followed by self-esteem (r = 0.47, *p* < 0.001) and prosociality (r = 0.36, *p* < 0.001). Intercorrelations among the socioemotional competencies were moderate in magnitude (r range = 0.28–0.41), supporting their conceptual distinctiveness and justifying their joint inclusion in subsequent multivariate analyses.

### 3.2. Distribution of Socioemotional Competency Profiles

To facilitate interpretability, socioemotional competencies were classified into low, moderate, and high levels using percentile-based criteria (≤25th percentile = low; 26th–74th percentile = moderate; ≥75th percentile = high).

As displayed in Table 2, the majority of teachers exhibited moderate levels of self-esteem (58%), prosociality (64%), and emotional autonomy (41%). However, notable proportions of participants reported low levels of emotional autonomy (33%) and prosociality (26%), suggesting substantial heterogeneity in these competencies within the teaching population.

Comparative examination of variability indicators revealed that emotional autonomy showed the greatest dispersion, consistent with its broader interquartile range. This pattern suggests that teachers’ emotional self-regulation and independence capacities vary more markedly than their global self-evaluations or interpersonal dispositions.

Overall, these findings indicate that most teachers show moderate socio-emotional competency profiles, although a non-negligible proportion presents low levels (particularly in emotional autonomy) suggesting meaningful variability within the teaching population. This distribution highlights the relevance of examining how these competencies may differentially relate to teachers’ attitudes toward inclusive education in subsequent analyses.

### 3.3. Differences According to Gender and Teaching Experience

Gender differences in socioemotional competencies were examined using independent-samples *t*-tests. As presented in Table 3, female teachers reported significantly higher levels of prosociality than male teachers, t(588) = 6.12, *p* < 0.001, with a moderate-to-large effect size (Cohen’s d = 0.62). Conversely, male teachers showed significantly higher self-esteem levels than female teachers, t(588) = −3.48, *p* < 0.001, although the effect size was small-to-moderate (d = 0.31). No statistically significant gender differences were observed for emotional autonomy (*p* = 0.256).

Differences according to teaching experience were examined using one-way analyses of variance across three professional stages (0–3 years, 4–19 years, and 20–36 years). Significant effects emerged for self-esteem, F(2, 587) = 18.47, *p* < 0.001, η^2^ = 0.059, and prosociality, F(2, 587) = 22.13, *p* < 0.001, η^2^ = 0.070. Post hoc analyses indicated that self-esteem increased progressively with professional experience, whereas prosociality was significantly higher among early-career teachers and declined across subsequent career stages. No significant differences were found for emotional autonomy.

Regarding attitudes toward inclusive education, no gender differences were identified. However, a significant effect of teaching experience was observed, F(2, 587) = 16.92, *p* < 0.001, η^2^ = 0.055, with more favorable attitudes among teachers with fewer years of experience and lower levels among those with longer professional trajectories.

The analyses showed some statistically significant differences in specific socio-emotional competencies according to gender and teaching experience. Female teachers reported higher prosociality, and male teachers showed slightly higher self-esteem, while no gender differences were observed for emotional autonomy or inclusive attitudes. Regarding teaching experience, significant differences emerged for inclusive attitudes, with higher levels at the beginning of teachers’ careers. These results describe the distribution of competencies and attitudes across groups and provide contextual information for the subsequent predictive analyses.

### 3.4. Associations Between Socioemotional Competencies in Attitudes Toward Inclusive Education

A multiple linear regression analysis was conducted to examine the extent to which socioemotional competencies were statistically associated with teachers’ attitudes toward inclusive education.

The overall model was statistically significant, F(3, 586) = 83.42, *p* < 0.001, explaining 29.9% of the variance in inclusive attitudes (adjusted R^2^ = 0.295). As shown in Table 4, all predictors made significant independent contributions to the model.

Emotional autonomy showed the strongest statistical association with inclusive attitudes among the competencies examined (β = 0.443, *p* < 0.001), uniquely accounting for approximately 11.4% of the explained variance. Self-esteem also showed a substantial contribution (β = 0.348, *p* < 0.001), followed by prosociality (β = 0.232, *p* < 0.001). These findings indicate that teachers’ internal emotional self-regulation capacities play a more central role in shaping inclusive attitudes than interpersonal prosocial tendencies alone.

## 4. Discussion

The present study sought to examine the relationship between primary school teachers’ socioemotional competencies and their attitudes toward inclusive education, with a particular focus on self-esteem, prosociality, and emotional autonomy. Overall, the findings provide robust empirical support for the central role of teachers’ internal emotional resources in shaping inclusive attitudes, extending previous research that has primarily emphasized structural, contextual, or training-related factors.

First, the descriptive results indicated that most teachers displayed moderate levels of socioemotional competencies. While this pattern is consistent with prior studies conducted in the Spanish and European contexts (Llorent & Núñez-Flores, 2023; Padilla, 2021), the observed variability (especially in emotional autonomy) suggests that teachers’ emotional development is far from homogeneous. This heterogeneity is particularly relevant in inclusive education, where emotional self-regulation, tolerance of uncertainty, and adaptive coping strategies are continuously required. The presence of a substantial proportion of teachers with low emotional autonomy may therefore represent a latent vulnerability factor for the sustainability of inclusive practices. Regarding group differences, the finding that women reported higher prosociality aligns with the extensive literature documenting gender-based socialization patterns in helping and empathic behaviors (Gómez Yepes et al., 2024; Navarro-Mateu et al., 2019; Sánchez-Pujalte et al., 2021). Conversely, the higher self-esteem observed among male teachers is consistent with meta-analytic evidence indicating persistent gender differences in global self-evaluative beliefs across adulthood. Importantly, however, these differences did not translate into differential attitudes toward inclusive education, reinforcing the notion that inclusive attitudes are not merely a function of demographic characteristics but rather of deeper psychological competencies.

The analyses according to teaching experience revealed a particularly noteworthy pattern. While self-esteem increased with professional experience, both prosociality and attitudes toward inclusive education declined over time. This apparent paradox suggests that professional confidence alone may not be sufficient to sustain inclusive commitment throughout a teaching career. One plausible explanation lies in the cumulative emotional demands associated with prolonged exposure to heterogeneous classrooms, institutional constraints, and perceived lack of support. Over time, these factors may contribute to emotional fatigue, which, in turn, undermines teachers’ willingness to engage in inclusive practices despite increased professional self-assurance. This interpretation is consistent with research linking long-term teaching experience to elevated burnout risk in inclusive contexts (Candeias et al., 2021).

The core contribution of the present study lies in the predictive model examining the role of socioemotional competencies in attitudes toward inclusive education. The results demonstrated that emotional autonomy emerged as the strongest predictor, surpassing both self-esteem and prosociality. This finding offers an important conceptual clarification. Whereas prosociality reflects interpersonal motivation and moral orientation, and self-esteem reflects general self-evaluation, emotional autonomy represents the capacity to regulate emotions independently, maintain emotional balance under pressure, and act in accordance with internal values rather than external demands. From this perspective, emotional autonomy may function as a psychological buffer that enables teachers to navigate the emotional complexity inherent in inclusive classrooms. Teachers with higher emotional autonomy are likely better equipped to manage frustration, uncertainty, and perceived inefficacy, allowing them to remain aligned with inclusive principles even under challenging conditions (Gómez Yepes et al., 2024). This interpretation is consistent with theoretical models emphasizing emotional self-regulation as a cornerstone of professional resilience in education (Calandri et al., 2025). Self-esteem also contributed substantially to inclusive attitudes, supporting previous research linking positive self-concept and perceived competence to openness toward educational innovation and diversity (Gkouvousi et al., 2024). Teachers with higher self-esteem may perceive inclusive education not as a threat to their professional identity but as a manageable challenge, thereby fostering greater acceptance and engagement. Prosociality, while statistically significant, displayed a comparatively smaller predictive weight. This finding suggests that altruistic dispositions alone may be insufficient to sustain inclusive attitudes in the absence of strong internal emotional resources (Albalá Genol et al., 2023). In other words, teachers may be highly motivated to support diverse learners, yet without adequate emotional autonomy or confidence, this motivation may not translate into enduring positive attitudes. This nuanced distinction contributes to ongoing debates in inclusive education research, which increasingly emphasize the need to move beyond purely value-based explanations. Taken together, the findings underscore the importance of conceptualizing inclusive attitudes as the product of both interpersonal values and intrapersonal regulatory processes. Inclusive education, therefore, should not be understood solely as a pedagogical or ethical endeavor but also as an emotionally demanding professional practice requiring advanced socioemotional competencies. From a practical standpoint, these results carry significant implications for teacher education and professional development.

Traditional training programs have largely prioritized methodological, curricular, and organizational competencies, often assuming that emotional skills are either innate or secondary. The present findings challenge this assumption by demonstrating that emotional autonomy, in particular, plays a decisive role in fostering inclusive attitudes. Consequently, teacher education programs should systematically incorporate evidence-based emotional competence training aimed at strengthening self-regulation, emotional awareness, and autonomous decision-making. Thus, the present study contributes to the growing body of evidence highlighting teachers’ emotional development as a central mechanism in inclusive education. By demonstrating the differential predictive roles of emotional autonomy, self-esteem, and prosociality, this research advances the current understanding of the psychological foundations underlying inclusive attitudes and offers a more nuanced framework for supporting teachers in increasingly diverse educational environments.

In particular, the prominent role of emotional autonomy observed in this study highlights the need for teacher education programs (both initial and in-service) to move beyond general awareness of inclusion and incorporate structured training in emotional regulation and professional resilience. Such training may include strategies for managing classroom-related stress, reflective practices aimed at strengthening autonomous decision-making in emotionally demanding situations, and supervised experiential activities that help teachers develop adaptive coping responses when facing diverse learning needs. By fostering teachers’ capacity to regulate emotions independently and sustain balanced responses under pressure, these approaches may contribute to more stable and enduring inclusive attitudes and practices in everyday classroom contexts (Calandri et al., 2025).

## 5. Conclusions

The findings of this study indicate that inclusive education depends not only on material resources or regulatory frameworks but also on teachers’ personal and emotional development. Inclusive attitudes emerge from the interaction between professional competencies and socio-emotional competencies, particularly emotional autonomy, self-esteem, and prosociality.

In line with international perspectives emphasizing the need to transform teachers’ beliefs, attitudes, and practices to advance toward full inclusion (Ainscow, 2020), the present results suggest that strengthening teachers’ socio-emotional competencies represents a key pathway for promoting high-quality inclusive education. Investing in teachers’ emotional well-being and personal development ultimately constitutes an investment in more equitable, resilient, and inclusive educational systems.

Finally, several limitations should be acknowledged, leading us to propose some avenues for future research. Longitudinal studies are needed to examine whether enhancements in socioemotional competencies lead to sustained changes in inclusive attitudes over time. Second, the reliance on self-report measures may introduce social desirability bias. Future research should incorporate observational indicators of inclusive practices and multi-informant assessment strategies. In addition, intervention-based studies aimed at strengthening competencies such as emotional autonomy, self-esteem, and prosociality would be particularly valuable to determine whether targeted training programs can effectively promote more inclusive attitudes and practices. Finally, although the sample size was substantial, participants were drawn from a single Spanish region, which may limit generalizability to other sociocultural contexts.

Despite the limitations acknowledged and the improvements suggested for future research, the present study provides meaningful findings for understanding and enhancing teachers’ inclusive attitudes as shaped by socio-emotional competencies. In particular, the results highlight the relevance of emotional autonomy, self-esteem, and prosociality as key psychological resources supporting more favorable orientations toward inclusive education, offering a useful basis for both future research and teacher training initiatives.

## Figures and Tables

**Table 1:** Descriptive statistics and Pearson correlations among study variables.

	M	SD	1	2	3	4
1. Self-esteem	27.84	4.96	-			
2. Prosociality	128.21	18.73	0.34 ***	-		
3. Emotional autonomy	57.46	10.12	0.41 ***	0.28 ***	-	
4. Inclusive attitudes	3.71	0.62	0.47 ***	0.36 ***	0.52 ***	-

**Note.** M: Mean; SD = standard deviation. ***, *p* < 0.001.

**Table 2:** Distribution of socioemotional competency levels.

Competency	Low (%)	Moderate (%)	High (%)
Self-esteem	15	58	27
Prosociality	26	64	10
Emotional autonomy	33	41	26

**Table 3:** Group differences by gender and teaching experience.

	Group	M	SD	Test	*p*	Effect Size
Prosociality	Women	131.02	17.84	*t*	<0.001	*d* = 0.62
	Men	119.47	19.62			
Self-esteem	Women	27.62	4.98	*t*	<0.001	*d* = 0.31
	Men	29.12	4.71			
Inclusive attitudes	0–3 years	3.92	0.58	ANOVA	<0.001	*η*^2^ = 0.055
	4–19 years	3.69	0.61			
	20–36 years	3.42	0.66			

**Table 4:** Multiple linear regression predicting attitudes toward inclusive education.

Dependent Variable	Independent Variables	Β (95% CI)	S.E.	β	*t*	∆R^2^
Attitudes Toward Inclusive Education	Self-esteem	0.484	0.099	0.348	4.902 ***	0.299
	Prosociality	0.296	0.100	0.232	2.965 ***	
	Emotional autonomy	0.597	0.103	0.443	5.803 ***	

**Note.** B: unstandardized coefficient; S.E. = standard error; β = standardized coefficient. ***, *p* < 0.001.

## Data Availability

The raw data supporting the conclusions of this article will be made available by the authors upon request.
